# Wear of feldspathic-ceramic-veneered zirconia posterior FPDs after 10 years

**DOI:** 10.1186/s12903-020-01336-8

**Published:** 2020-11-30

**Authors:** Ragai-Edward Matta, Constantin Motel, Elena Kirchner, Simon Paul Stelzer, Werner Adler, Manfred Wichmann, Lara Berger

**Affiliations:** 1 Department of Prosthodontics, Erlangen University Hospital, Glueckstrasse 11, 91054 Erlangen, Germany; 2 Zahnarztpraxis Haidhausen Dr. Hans-Rudolf Kurpiers und Christian Pollok, Weißenburger Platz 8, 81667 Munich, Germany; 3 Department of Medical Informatics, Biometry and Epidemiology, Friedrich-Alexander-University of Erlangen Nuremberg, Waldstr. 6, 91054 Erlangen, Germany

**Keywords:** Wear of zirconia, Abrasive behavior of all-ceramics, Ceramic bridges, Occlusal interactions, Zirconia ceramics

## Abstract

**Background:**

The abrasion behavior of various ceramics is rarely investigated, though it is relevant for the clinical success of such restorations. The aim of this in vivo study was to evaluate the wear of feldspathic-ceramic-veneered zirconium oxide frameworks over a period of at least 10 years.

**Methods:**

The abrasion behavior of 15 bridge constructions from 15 different participants was examined after a period of 3, 5, and 10 years using plaster models, which were then subjected to a scanning process on the Atos II industrial scanner and digitized for three-dimensional evaluation of the abrasion by the corresponding software (ATOS Professional 7.6). The individual post-examination models were compared to the baseline model and deviations calculated in the sense of the largest, punctual loss of material in millimeters (“minimal distance”), the average abrasion in millimeters (“mean distance”), and the volume decrease in cubic millimeters (“integrated distance”). Statistical analyses were performed using the Wilcoxon sign rank test or mixed regression models. Multiple testing was considered by Benjamini-Hochberg correction. The significance level was set at 0.05.

**Results:**

We found steadily increasing wear of the ceramic. The average volume decrease was significant (*P* < 0.001) at 3 years and 10 years (− 3.25 mm^3^ and − 8.11 mm^3^, respectively).

**Conclusions:**

The results of this study indicate that the rate of volume loss in feldspathic-ceramic-veneered zirconia frameworks in the posterior region increases significantly over time. An increasing frequency of parameters was observed, particularly in the second half of the study period. However, the use of this class of materials can be considered clinically acceptable.

*Trial registration* This study is registered in DRKS - German Clinical Trials Register with the register number DRKS00021743. https://www.drks.de/drks_web/navigate.do?navigationId=trial.HTML&TRIAL_ID=DRKS00021743

## Background

Loss of hard tooth substance of the human dentition represents a natural wear phenomenon that increases in severity with increasing age [1]. Accordingly, special attention should be paid to the abrasive behavior of a dental material with the aim of finding an optimum between its own resistance to wear and low abrasiveness to the antagonist, and a wear comparable to that of natural teeth, which is a major challenge for current research [2]. Wear is generally considered to be the result of occlusal interactions, and the decision on supposedly suitable dental material has a direct effect on the antagonist. Both the restorative material and the antagonist can be severely abraded. In addition, chewing function may be impaired, resulting in a reduced quality of life and possible deterioration of systemic health [3, 4]. Finally, the susceptibility of the materials to wear could also affect the durability of the corresponding dental prosthesis [5].

With regard to the wear of a dental restoration and the resulting interactions with antagonists, we must take into account the different structures and mechanical properties of the materials used, as well as the tooth structure, because they influence the degree of wear. For example, tooth enamel exhibits a different wear behavior than dentin because they differ in their structural composition [3]. In contrast, the abrasion of a gold alloy corresponds approximately to that of natural tooth enamel, whereas non-precious metal alloys are mechanically stiffer due to their increased strength, resulting in a lower wear rate [6–8]. An amalgam alloy has the ability to deform, which is why it is generally considered wear-resistant [5]. In addition, a ceramic must be smooth and polished because a rough surface would lead to increased wear of both natural teeth and other restorative materials [3, 9]. Therefore, factors that influence the general abrasion behavior include the hardness, the microstructure, and the surface quality of a material [9]. Wear can also be caused by individual patient-related conditions, such as bruxism, traumatic tooth brushing, or reduced salivation, illustrating the need for proper selection of a suitable restorative material in the course of treatment planning and careful monitoring of the restoration in routine dental care [3].

Due to permanent improvements in the material properties and high aesthetic demands of patients, all-ceramics has become indispensable in everyday clinical practice as a material for restorations [10]. In light of the growing demand for highly aesthetic restorations, it is the dentist’s task to both keep up to date with the latest scientific research and to develop scientific evidence to support the clinical application of materials [11, 12]. Special attention has been paid to zirconium dioxide as a dental material due to its superior mechanical properties, high biocompatibility, and natural appearance [13–15]. Because of its good mechanical strength, zirconium dioxide can be used as a framework material for all-ceramic fixed dentures [16–18]. However, with regard to aesthetic requirements, the manufactured frameworks need a suitable veneering ceramic, which is the weakest link in the entire construction [19–21]. In contrast, more recent developments, as another step in CAD/CAM (Computer-aided design/ Computer-aided manufacturing) technology, present monolithic zirconia as a restorative material, which is intended to be used for the fully anatomical reconstruction of dentures without a veneer. Currently, clinical studies and scientific knowledge of the properties, prognosis, and long-term survival of this material are rare [22].

In this context, a 2019 study compared fixed FPDs (fixed partial dentures) made of zirconium oxide and fabricated with the IPS e.max ZirCAD system to metal-ceramic dentures over a period of 5 years. Both material classes exhibited a survival rate of 100%; thus, zirconium dioxide can be considered a potential alternative to a metal-ceramic restoration [23]. This was also confirmed by Teichmann et al., who evaluated three- to six-unit bridge constructions made of veneered zirconium oxide frameworks over an observation period of 10 years. The survival rate was still 95%, which was also reflected in the high satisfaction of the patients. This supports the use of zirconium oxide-based restorations as a reliable material in everyday clinical practice [24]. In another study, the long-term survival of three-unit fixed restorations made of a glass-infiltrated zirconia-reinforced alumina ceramic (In-Ceram Zirconia) was 93.6% after a mean observation period of 9.7 years [25]. The study by Kern et al. can be used to evaluate the long-term behavior of all-ceramic restorations. The survival rate of FPDs made of a monolithic lithium disilicate ceramic was 87.9% after a mean period of 121 months, which also confirms the use of this material as an alternative to metal-ceramic restorations [26].

However, this class of materials also has technical limitations, particularly the chipping of the veneering ceramic. On the other hand, fractures in the framework occur rarely, but a general cumulative increase in the chipping rate can be observed [23]. This can amount to 10% after 5 years in clinical use of zirconium oxide-based restorations, whereas the chipping rate can double after another 5 years. Chipping has been observed in 52.7% of cases after 11 years [24].

The detailed reproduction and adjustment of surfaces can be achieved by light-optical precision scanning methods, by which digital and three-dimensional (3D) information can be obtained using special computer software after scanning an object surface [27–29]. This very promising technique for 3D object registration and data acquisition is enabled by innovative non-contact scanners that are already established in industry and represent a routine application [30]. This principle, which is also used in our study, has very high precision with small discrepancies [31].

This study presents a method for visualizing abrasion behavior on the basis of three- and four-unit all-ceramic bridges in the posterior region made of feldspathic-ceramic-veneered Lava™ Frame zirconium dioxide frameworks (Lava™ system, 3 M) in clinical use for 10 to 12 years at the time of investigation.

## Methods

As part of the clinical study, which was approved in advance by the ethics committee of the University of Erlangen (Reference number: 2832), the abrasive behavior of all-ceramic zirconium dioxide bridges veneered with feldspathic-ceramic was investigated at Dental Clinic 2 - Department of Dental Prosthetics of Friedrich-Alexander-University Erlangen-Nuremberg, Erlangen, Germany, after a minimum observation period of 10 years using a light-optical scanning procedure with a 3D industrial scanner.

The manufacturing process of all prosthetic restorations was standardized and carried out by the same technician at the same milling center. He milled the framework (LAVA™ Frame, 3 M), which was composed of pre-sintered 3-yttria-stabilized zirconia blanks with a grain size of 0.5 μm, and finally sintered the zirconia framework using CAD/CAM technology. After an intraoral fitting of the restorations, a specially adapted veneering (LAVA™ Ceram, 3 M) was applied according to the manufacturer’s recommendations. In the final step, the framework was coated with a glaze after another fitting and fired.

The optical 3D measurements and evaluations were based on conventional silicone impressions (AFFINIS heavy body +AFFINIS light body, Coltène/Whaledent GmbH & Co KG, Altstätten, Switzerland) made in 15 subjects with one bridge construction each at the beginning of the study period and after 36, 60, and 120 months, as well as the subsequent hard gypsum models (Fujijock, Multident Dental, Oldenburg, Germany), which were digitized using a 3D white light scanner (ATOS II, GOM GmbH, Brunswick, Germany) and the corresponding evaluation software (ATOS Professional 7.6, GOM GmbH, Brunswick, Germany) in order to compare the abrasive behaviors. Only three- or four-unit restorations of subjects who appeared for impressions after a telephone contact at each of the indicated examinations were included in the statistical analysis. In the maxilla, 7 restorations were examined, consisting of 10 premolar and 14 M bridge components. In the mandible, the abrasion of 11 premolar and 15 M parts in 7 bridge constructions was evaluated. This resulted in evaluating wear behavior in a total of 50 bridge pontics.

The plaster models were subsequently equipped with prefabricated reference points (diameter: 0.8 mm), provided by the company GOM (GOM GmbH, Brunswick, Germany), in preparation for digitization. These reference points served as support points for the scanning software to transform the individual images into a complete 3D model. The clear constellation of the GOM glue points ensured the correct position of the scanned surfaces in the coordinate system and guaranteed the accuracy of this process. The individual measurement data required for optimal representation of the object were saved in the form of a so-called point cloud, though the digital 3D model had to have a closed surface in order to carry out the measurements. This was made possible in the software by using polygons, resulting in a polygon mesh in the form of a Surface Tessellation/Triangulation Language (STL) file [32]. Thus, a digital representation of the four plaster models was created for each test person from the impressions taken during the follow-up examinations.

In the next step, the individual post-examination models (36 months, 60 months, 120 months) were “matched” with the corresponding baseline model as a reference in order to compare the occlusal material loss, which indicated the abrasion of the ceramic over time, to the bridges after 3, 5, and 10 years of wear. A manual 3-point alignment of the models was performed to obtain the initially required rough pre-alignment. The tools used to implement this measure were striking points on both objects, such as prominent features on anterior or canine teeth, which were manually marked as precisely as possible on both the baseline and the corresponding virtual model.

Possible inaccuracies in the plaster were to be avoided during the analysis of abrasion; therefore, the area to be measured, which corresponded to the all-ceramic restoration on the baseline models, was selected over the surface, guaranteeing continuous constancy of the area to be measured with regard to the individual evaluations of the different years. At this point, a distinction must be made between complete selection of the occlusal surface of the bridges in the first test set-up and a separate selection of the individual bridge anchors and pontics (premolars and molars, respectively). In the last step of the “matching” process, the “local best-fit” option was used to achieve the final alignment by optimally aligning the selected sections with each other on the models using the best-fit function enabled by the software. Thus, on the basis of the selected surfaces, possible deviations in the restorations between the baseline model and the reference of the corresponding year could be investigated.

The associated evaluation software (GOM Professional 7.6, GOM GmbH, Brunswick, Germany) calculated the volume difference between the respective models and enabled the presentation of changes in the measuring objects to be compared based on a color gradient located on the occlusal surface of the digitalized model. The false-color image illustrated both a decrease in volume resulting from wear processes (negative deviations marked by blue areas) and an increase in the existing volume (positive differences marked by red areas), which could be explained by potential sources of error within the impressions or the plaster models created from them. A constant volume of both models in terms of relative agreement were marked as green areas.

The values calculated using the ATOS II industrial scanner and the corresponding software for the analysis of the abrasion behavior of the ceramic restorations included the largest punctual material loss in millimeters (“minimum distance”), the average removal of the surface in millimeters (“mean distance”), and the volume reduction of the all-ceramic restoration in cubic millimeters (“integrated distance”).

The statistical programming language R V 3.6.3 (R Core Team (2020), R Foundation for Statistical Computing, Vienna, Austria) was used for all statistical analyses. In the first test set-up described above, Wilcoxon sign rank tests were conducted. In the second experimental set-up, the premolar/molar comparisons were performed using mixed regression models. To account for multiple testing, *P* values were corrected using the Benjamini-Hochberg method. The level of significance was set to 0.05.

## Results

Based on the 3D measurements of the entire occlusal surface of the restorations using industrial scanning technology in the first experimental set-up, the average minimum distance after 3 years was − 210 μm and after 120 months was − 640 μm. The tabular compilation of the calculated *P*-values in relation to minimum distance were compared in relation to the period of the follow-up examinations (36, 60, and 120 months), and each of the P-values was significant.

In relation to the minimum distance of the select pontics, after 3 years, − 132 μm was the largest occlusal loss of ceramic with regard to the premolars, whereas a significantly higher − 251 μm was measured for the molars. At the time of the last follow-up examination, − 278 μm was documented for the premolars and − 435 μm for the molars. The results illustrate a different abrasive behavior of the premolar and molar components of dental bridges, with continuously higher values of calculated material loss recorded for the molars.

In addition, the mean distance of the complete occlusal surface of the bridges was determined to be a mean loss of − 48 μm after 36 months. An ongoing increase in material wear was indicated by the greatest average wear of the ceramic being measured at 120 months, with a significant − 131 μm.

For the mean distance for the selection of the individual bridge segments, an average ceramic removal of − 38 μm was recorded after 36 months for the premolars and − 46 μ, for the molars. The average abrasion of the restorations after 10 years was − 79 μm with regard to the premolars and − 117 μm with regard to the molar areas of the bridges, which ultimately showed remarkably greater material wear of the molars at that follow-up.

The integrated distance, as a representative of the average volume decrease, was − 3.25 mm^3^ after 36 months. The significant measurement of − 8.11 mm^3^ after 120 months demonstrated increasing loss of volume over time.

In contrast to selection of the complete occlusal surfaces of the feldspathic-ceramic-veneered all-ceramic bridges, in the second test set-up, each individual bridge pontic was selected separately via the surface. Thus, the integrated distance of the selected individual bridge components was determined in the second test set-up; after 3 years of wear, the average reduction in the volume of the premolars and molars was − 0.539 mm^3^ and − 1.269 mm^3^, respectively. Starting from the time the restorations were placed, after 120 months an average decrease in volume of − 2.282 mm^3^ for the premolars, and finally − 3.253 mm^3^ for the molars, was recorded. Thus, the wear of the ceramic material, which increased steadily over time, was greater in the second half of our study than in the first 5 years of the observation period.

**Fig. 1:**
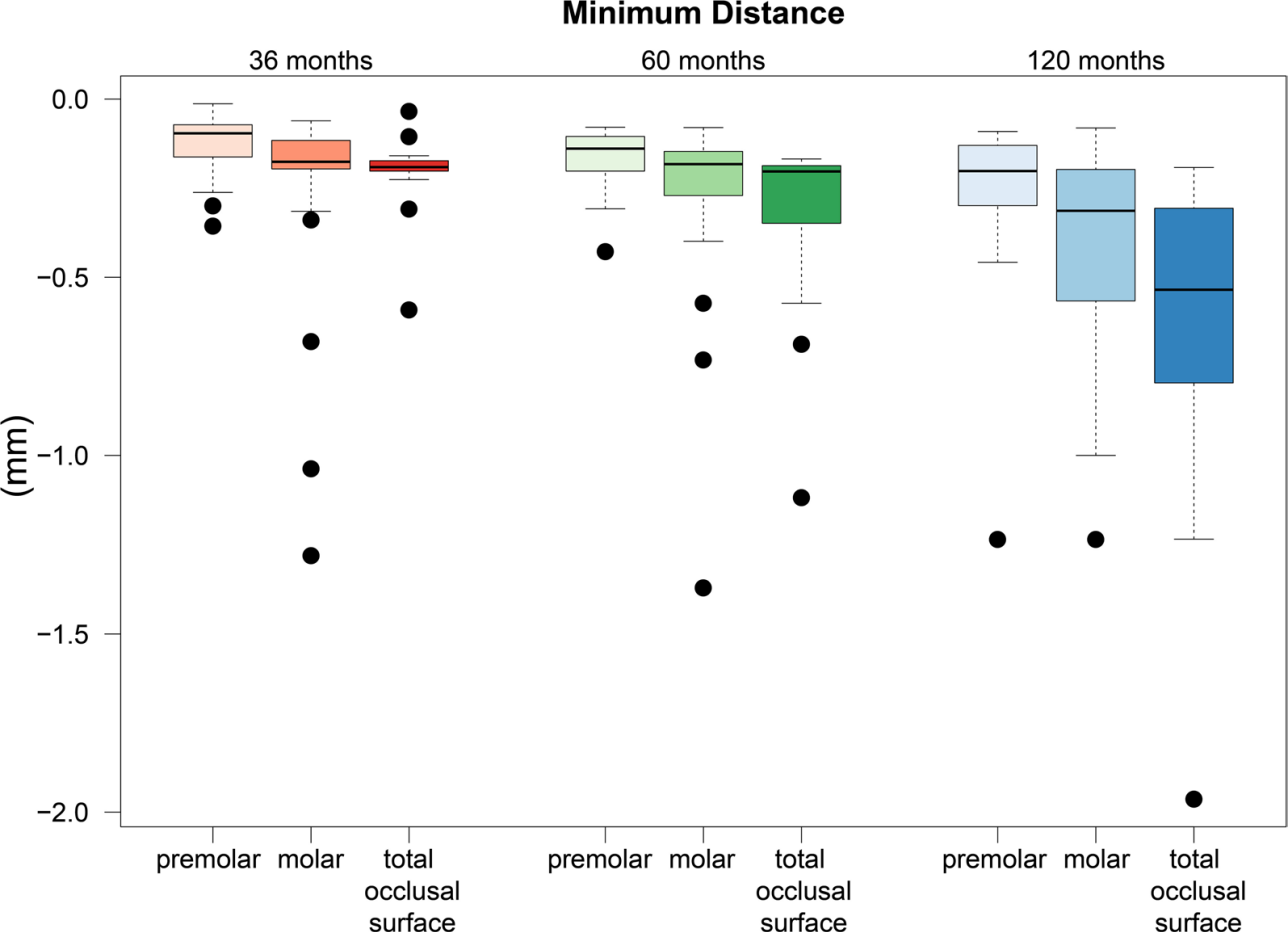
Punctual material loss after 3, 5, and 10 years

**Fig. 2:**
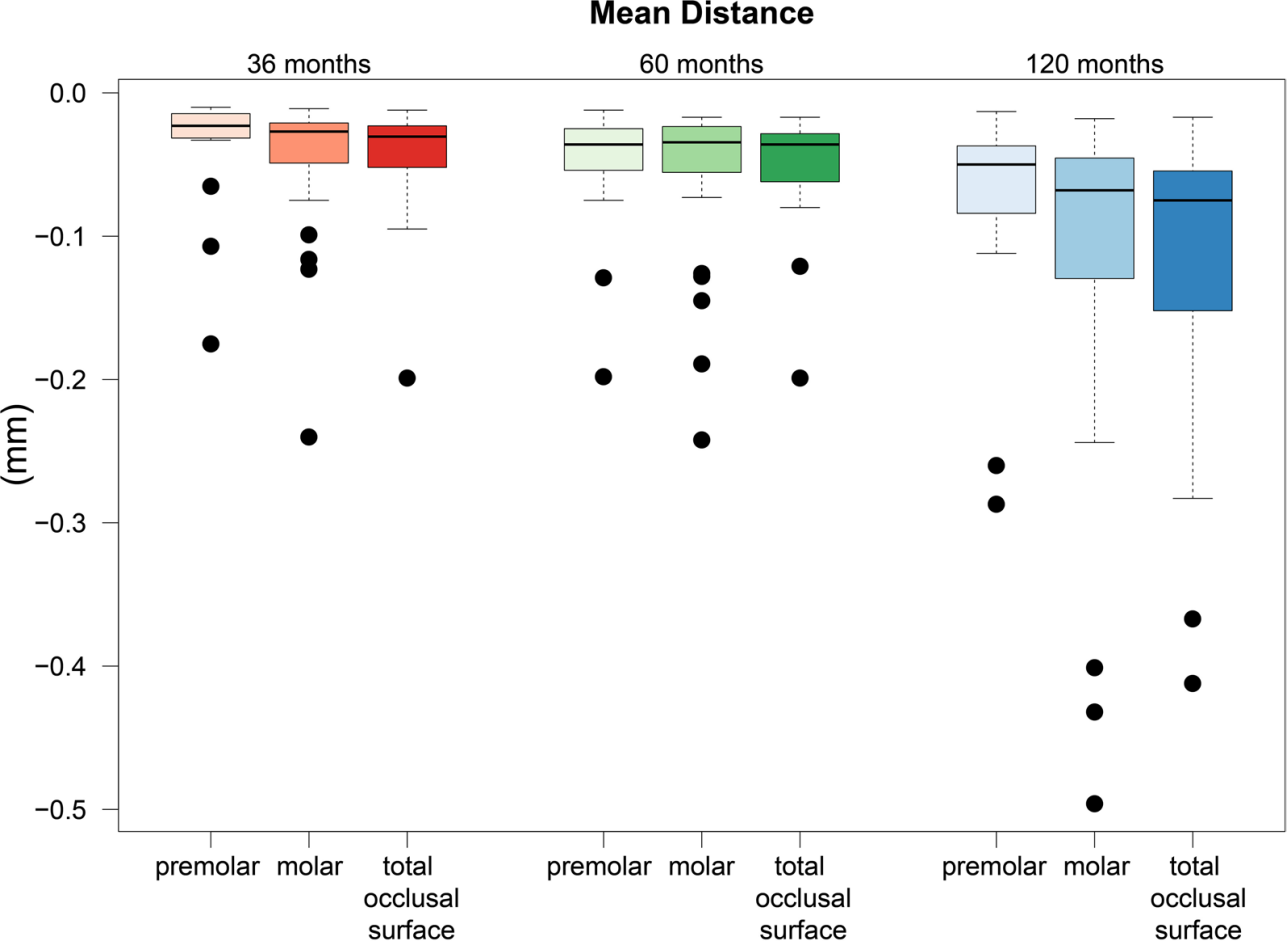
Average removal of the surface to assess the abrasive behavior

**Fig. 3:**
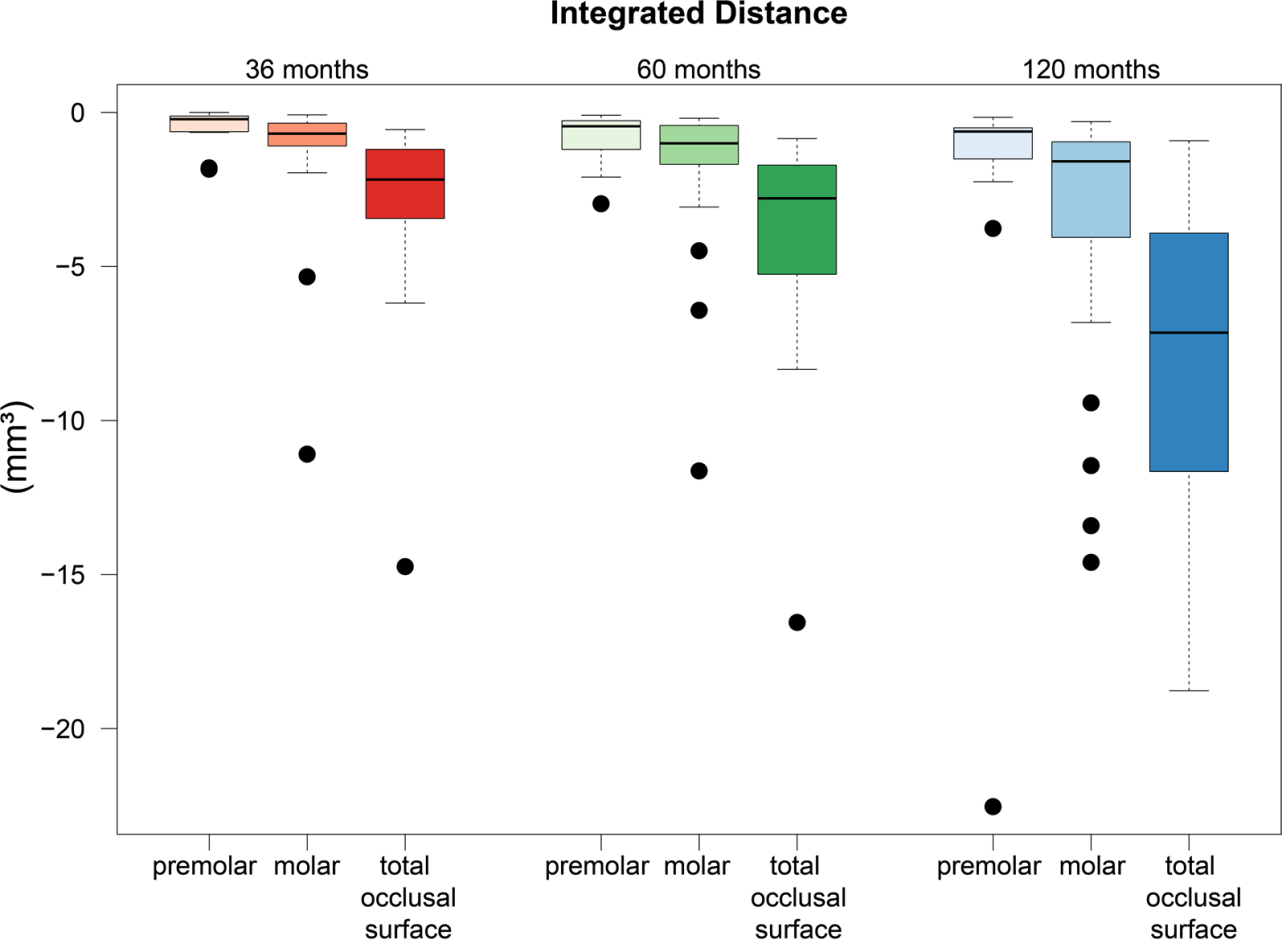
Volume reduction over 10 years

**Fig. 4:**
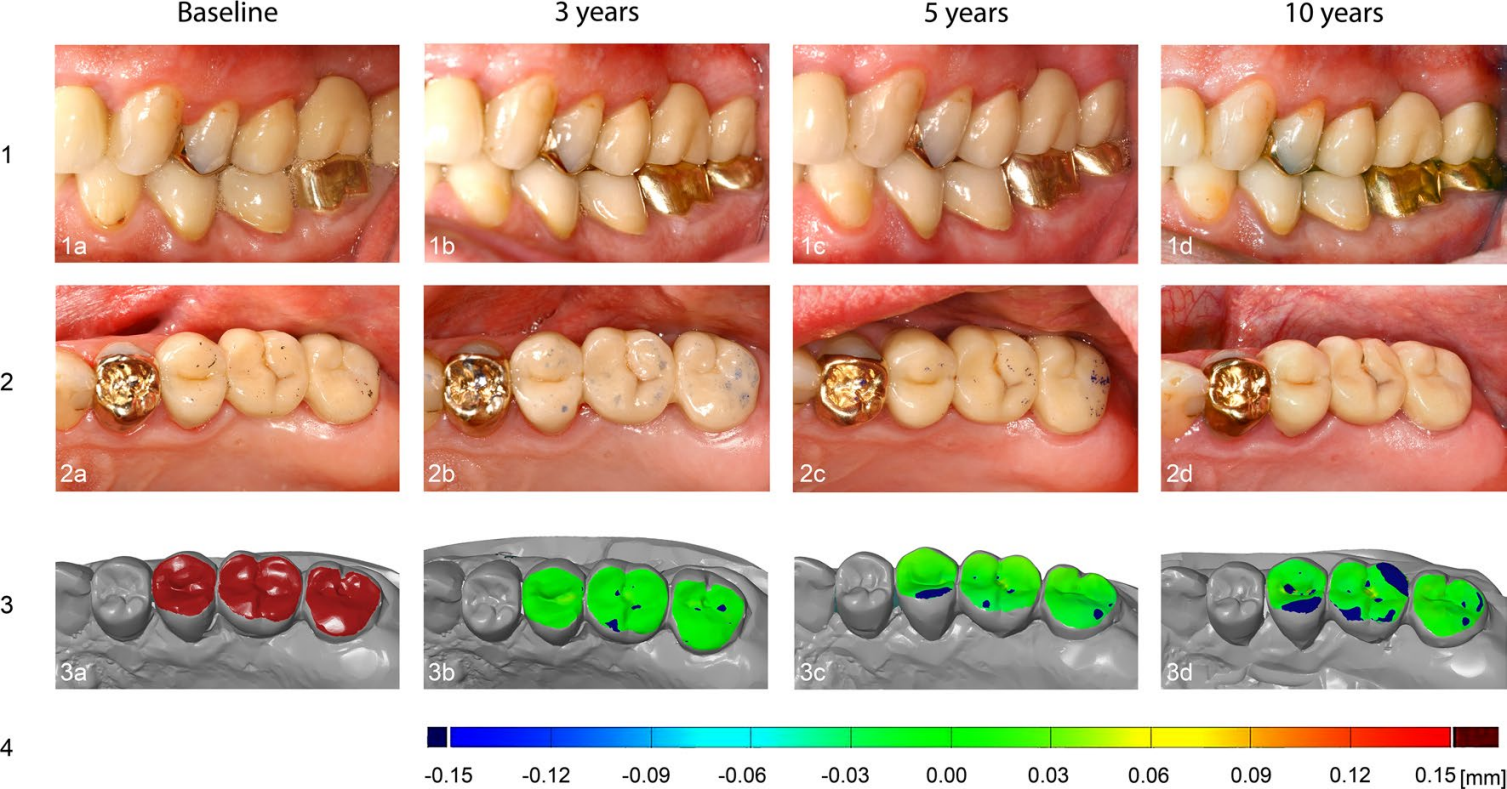
Clinical and virtual 3D representation of the wear of a restoration. Series 1: Vestibular view. Series 2: Occlusal view. Series 3: 3D view after digitization of the plaster models. a, selected area; b-d, false color display in which the abrasive areas are marked in blue. Green areas have had no or almost no abrasive process. Series 4: false color scale

The results of the numerical evaluation of the abrasive behavior of the restorative material are graphically presented using boxplot diagrams in Figs. 1, 2, 3. A color-coded illustrative presentation of the results is shown in Fig. 4. In addition, the respective materials of the antagonists of all 15 restorations examined on the corresponding examination dates are listed in Table 1.

**Table 1:** Proportion of antagonist materials at each evaluation. Data are presented as the proportion of all materials

Antagonist Material	Baseline	3 years	5 years	10 years
*Gold inlay*	13.5%	11.5%	11.5%	11.5%
*Gold crown*	19.2%	23.0%	23.0%	15.4%
*Ceramic inlay*	0%	0%	3.9%	17.3%
*Ceramic crown*	0%	5.8%	5.8%	5.8%
*Veneered metal crown*	11.5%	11.5%	15.4%	17.3%
*Amalgam filling*	26.9%	19.2%	11.5%	3.9%
*Composite filling*	9.6%	13.5%	15.4%	7.7%
*Acrylic denture tooth*	0%	0%	0%	7.7%
*Natural tooth*	17.4%	15.5%	13.5%	11.5%
*Missing antagonist*	1.9%	0%	0%	1.9%

## Discussion

Selection of the correct restorative material is fundamental to ensure both normal function and occlusal harmony [9]. A natural phenomenon is represented by the gradual abrasion in the dentition, and this process can be disturbed by the use of restorative materials to replace natural tooth structure [33]. Ultimately, the non-uniform structures and physical aspects between natural teeth and restorative materials result in different degrees of wear [3]. A number of studies have evaluated the long-term clinical behavior of ceramics [24, 34, 35], but studies on the loss of the vertical dimension are limited [36].

For this reason, we carried out the 3D light-optical examination of all-ceramic bridges using the Atos II scanning unit. The abrasive behavior was analyzed on the basis of various parameters using digitized virtual models corresponding to the condition of the dental restorations at the time of insertion and after 3, 5, and at least 10 years. Notably, both a decrease in volume due to wear processes over time and an increase in volume in some places was noted. This phenomenon could be caused by errors that occurred when taking the impression, such as insufficient adhesion of the impression to the impression tray or the localization of relevant areas outside its boundary [37], which were then carried over to the plaster models. Accordingly, to avoid falsification of the measurement results, the value range within the evaluations was adjusted, and the regions with an erroneous increase in volume were excluded. Alternatively, instead of a conventional impression, the use of innovative technology, such as an intraoral scanner, can be considered to take a digital impression. Current studies have reported that digital acquisition of intraoral information is at least comparable to the conventional method, and could even be more precise [38, 39]. However, digital impressions also entail technical limitations and system-specific deviations [39, 40].

Overall, an increasing and significant loss of material was characteristic of the all-ceramic bridge constructions during the entire investigation period, with the frequency of abrasion being higher in the second half of the investigation than in the first 5 years. This finding may be due to the loss of mechanical strength of dental ceramics over time. This could be caused by the different dissolution rates of the components, which could lead to increased surface roughness and accelerate the process of abrasion due to fatigue reactions [41]. Favorable factors here include permanent changes in the pH value in the oral cavity, which can vary between 1 and 10 depending on the nutrition, the drinks consumed, and the bacterial metabolic processes [42], as well as the considerable temperature fluctuations of up to 60° [41, 43]. The resulting roughness in the surface causes exposure of the filler particles and creation of cavities, which both increases plaque accumulation and contributes to enhancing wear [3]. Moreover, the roughness of the ceramic influences the susceptibility of the antagonistic natural tooth enamel to abrasion [44]. In addition, the results indicate that the premolar and molar bridge pontics did not react congruently to wear processes. In the case of the molars, higher individual values were recorded at each follow-up examination.

This was also observed in another in vivo study from 2008, in which crowns made of lithium disilicate were examined regarding their abrasion behavior by means of laser scanning the corresponding plaster models. After 1 year, the mean reduction in the occlusal volume of the crowns was 0.19 ± 0.06 mm^3^ for premolar restorations and 0.34 ± 0.08 mm^3^ for molar restorations [45]. Furthermore, this appearance is observed not only in the clinical use of dental ceramics, but also in the context of other material classes. For example, the average wear of metal-free polymer crowns after an observation period of 2 years after insertion on premolars and molars was 44 μm and 84 μm, respectively. Moreover, a significant dependence of the degree of wear on the molar or premolar crown localization was deter-mined [46]. Our results on the average loss of − 38 μm for the premolars and − 46 μm for the molars were also significantly lower after 3 years of examination. The reason for this could be the occlusal surface, which increases in size with the molar region, resulting in a more pronounced chewing force in the distal part of the jaw [47]. In addition, the occlusion has a significant effect on the process of wear [9].

The fact that abrasion in general is a progressive phenomenon [9] has also been confirmed by other studies that have dealt with the wear behavior of teeth and restorative materials. An example of this is the study by Mundhe et al., in which a comparable study design was used to investigate the wear of the natural, antagonistic tooth enamel in response to definitively cemented crowns in the opposing jaw on a ceramic and metal-ceramic basis in order to investigate the effects of a restoration material used in the oral cavity on the natural tooth enamel. The maximum linear wear was determined by means of plaster models obtained from impressions, which were subsequently digitized using a 3D white-light scanner. The results confirmed that significant wear occurred over time, though the investigation period was only 1 year [48]. Furthermore, a current in vivo study by Esquivel-Upshaw et al. used a 3D laser scanner to illustrate occlusal loss of material not only on monolithic zirconium and metal-ceramic crowns, but also on natural teeth. After 1 year, no significant differences were observed in the wear behavior of the different materials and the natural enamel [49]. In addition, a number of in vitro studies have evaluated the wear potential of various materials. For example, Zurek et al. recording the volume loss of zirconium and lithium disilicate ceramics after a chewing simulation using white-light interferometry as a non-contact, optical method of measurement and a scanning electron microscope. A significantly higher loss of material was recorded for the lithium disilicate samples, with a low abrasiveness of zirconium oxide [36]. D’Arcangelo et al. also carried out an in vitro investigation of the wear resistance of various ceramics under masticatory simulation against a test body made of zirconium oxide. The loss of vertical dimension and the volume decrease were recorded with a 3D scanner [50].

Basically, abrasion in the oral cavity usually results from tooth-to-tooth or tooth-to-restoration contact, and this process is generally accelerated by a dental prosthesis. Despite constant technical innovations in the context of current research, no valid in vivo method has been established to objectively assess abrasion behavior [51]. However, the procedure used in this study, the generation of virtual 3D models using the Atos II industrial scanner, proved to be a very practical method for neutrally investigating and displaying the wear behavior of all-ceramic restorations. Thus, our method could be regarded as a unified investigation method to create better comparability within different studies dealing with wear behavior. The results of the present study confirm that zirconia-based all-ceramic restorations are generally suitable for use as prosthetic treatment as described in similar studies [24, 52]. However, not only the wear of the material, but also abrasion of the antagonists caused by the surface interactions determine whether its use in everyday clinical practice can be justified. In this context, the antagonistic wear of enamel against monolithic zirconium dioxide crowns was evaluated in a recent review of the literature. The result was that the wear of the enamel by all-ceramic restorations was similar or greater than the interaction with natural teeth, though it was still less overall than for metal-ceramic restorations [53]. Furthermore, a clinical study was conducted to compare abrasion between monolithic zirconia crowns, natural antagonist teeth, and natural control teeth that were not faced with a restoration, but with natural tooth structure. The measurement was carried out via a laser scan of plaster models obtained from impressions. The mean vertical loss after 2 years was 46 μm for the enamel interacting with the ceramic, whereas the restoration had a mean abrasion of 14 μm. The values for the control teeth were between 19 and 26 μm [54]. The contrary was evaluated in another clinical study in which measurements were made using an intraoral digital impression technique, as significantly higher values were measured with approximate agreement. For enamel or ceramic antagonists, a maximum mean wear of 115 ± 71 μm and 120 ± 27 μm, respectively, was documented after 24 months [55]. These differences may be caused by differences in the surface condition, as this influences the wear behavior. For example, Kaizer et al. demonstrated that zirconia crowns polished in the pre-sintered state with occlusal glass infiltration are not only resistant to the wear phenomenon, but also gentle on the natural antagonist [56].

A comparison of the in vivo abrasive behavior with other classes of materials is difficult because most of the investigations were carried out in the laboratory and clinical data are hardly available, though clinical tests are essential to investigating the complex oral cavity [57, 58]. Furthermore, the correlation of data from in vitro studies evaluating abrasion with those from time-consuming clinical investigations is generally low [59], though different materials can still be evaluated comparatively under standardized conditions [60]. An example of a clinical study is the study by Ohlmann et al. in which the abrasion behavior of metal-free polymer crowns with and without a glass fiber framework in the posterior region was compared to the abrasion behavior of metal-ceramic crowns after 2 years using a 3D laser scanner. A mean occlusal wear of − 81.8 μm was measured for the glass-fiber-reinforced polymer-based restorations and − 76.8 μm for the polymer crowns without a glass-fiber framework, whereas the metal-ceramic restorations had a mean abrasion of − 38.5 μm. This suggests significantly higher abrasion of the polymer-based crowns than the metal-ceramic restorations [61]. A comparison to our results after 3 years of observation also shows that this class of materials exhibits a more pronounced abrasion behavior than all-ceramic restorations. In contrast, the degree of abrasion of the metal-ceramic restorations approaches our mean distance of the complete occlusal surface rate of 48 μm after 3 years.

It would also be possible to carry out measurements based on digital impressions of the restorations in future research, avoiding the conventional working step with the subsequent fabrication of a plaster model. In general, digital impression-taking can be considered an alternative to conventional methods, as they offer comparable accuracy [62]. In addition, impressions taken by conventional methods are generally associated with a high error rate, which is why the quality is unsatisfactory in many cases [37]. The precision of the plaster model is also influenced by a number of different factors, including the impression material, the tray material, the time interval between taking the impression and pouring, and the expansion of the plaster [63, 64].

Notably, the number of subjects undergoing in vivo diagnostics over a longer period of time is currently small [24, 52]. In our study, the abrasion behavior of a total of 15 restorations from 15 different participants was evaluated after 10 to 12 years. Long-term reports are required to obtain meaningful information about the clinical performance of a dental material [24].

## Conclusions

In this study, the wear of feldspathic-ceramic-veneered zirconium oxide frameworks used for bridge construction in the posterior region increased over time. The largest punctual loss of material in molars is subject to a stronger decrease after 10 years. In addition, the frequency of parameters used to examine the abrasion was higher in the last 5 years than in the first 5 years of the study period. Nevertheless, this class of materials has considerable advantages for use in dental restorations.

## Data Availability

The datasets used and/or analyzed during the current study are available from the corresponding author upon reasonable request.
